# Editorial: Golgi Pathology in Neurodegenerative Diseases

**DOI:** 10.3389/fnins.2015.00489

**Published:** 2016-01-06

**Authors:** Catherine Rabouille, Georg Haase

**Affiliations:** ^1^Hubrecht Institute of the KNAW (Royal Academy of Sciences) and UMC UtrechtUtrecht, Netherlands; ^2^Department of Cell Biology, UMC UtrechtUtrecht, Netherlands; ^3^Institut de Neurosciences de la Timone, Centre National de la Recherche Scientifique and Aix-Marseille Université UMR 7289Marseille, France

**Keywords:** Golgi apparatus, neurodegenerative disease, microtubules, vesicle trafficking, cell death, signaling, axon degeneration, cellular stress

The Golgi apparatus is a central organelle that lies at the heart of the secretory pathway sustaining the delivery of proteins from their site of synthesis in the endoplasmic reticulum to their final destination, the extracellular medium, the plasma membrane, and the endo-lysosomal system. It ensures post-translational protein modifications such as glycosylation and proteolytic cleavage and processing and acts as a sorting device including to neuronal axons and dendrites (Horton and Ehlers, 2003; Ye et al., 2007).

The mammalian Golgi apparatus was first described by Camillo Golgi in 1998 as “apparato reticolare interno,” “a fine and elegant network within the cell body … completely internal in the nerve cells” (Golgi, 1898a,b). This large reticulum comprises stacks of flattened membrane bound compartments called cisternae which are laterally linked to form the so-called Golgi ribbon.

Structural and functional alterations of the Golgi apparatus, which are here collectively termed Golgi pathology, are now recognized as a constant pathological hallmark of various neurodegenerative diseases including amyotrophic lateral sclerosis (ALS), Parkinson, Alzheimer, Huntington, and prion diseases (Fan et al., 2008). In ALS, structural Golgi alterations have been revealed by the pioneering work of Gonatas and colleagues (Mourelatos et al., 1990; Gonatas et al., 1992; Fujita et al., 2002). They manifest as fragmentation—transformation of the Golgi ribbon into disconnected stacks, cisternae, tubules and vesicles, and as atrophy—loss of Golgi membrane material.

These morphological changes are often accompanied by functional Golgi alterations, such as those affecting the anterograde and retrograde transport in the early secretory pathway, both in cellular models of Parkinson (Cooper et al., 2006; Cho et al., 2014), Huntington (Caviston et al., 2007; Pardo et al., 2010), and Alzheimer (Annaert et al., 1999; Joshi et al., 2014) diseases as well as in ALS (Stieber et al., 2004; Soo et al., 2015).

At least in ALS, Golgi pathology manifests as an early pre-clinical feature in degenerating neurons both in affected patients and in animal models (Mourelatos et al., 1996), suggesting that it may be relevant to the disease process instead of just representing an epiphenomenon. Yet, neither the molecular mechanisms underlying the changes in the functional organization of the Golgi apparatus nor their precise relevance to neurodegeneration have yet been completely elucidated.

These important questions got a new boost by the discovery of mutations in genes encoding Golgi-related proteins as direct causes of neurodegeneration. For instance, mutations in Optineurin (Maruyama et al., 2010), VPS54/wobbler (Schmitt-John et al., 2005), and TBCE/pmn (Martin et al., 2002) have been identified in ALS and related motor neuron diseases. Furthermore, mutations in the Parkinson disease-associated proteins α-Synuclein (Cooper et al., 2006; Thayanidhi et al., 2010), LRRK2 (Lin et al., 2009; Cho et al., 2014), Parkin (Shimura et al., 1999; Kubo et al., 2001), and VPS35 (McGough et al., 2014; Zavodszky et al., 2014; Malik et al., 2015) have been shown to affect Golgi structure or transport processes to and from the Golgi.

Furthermore, the recognition of Golgi-derived microtubules and their specific functions, the better understanding of Golgi transport processes, the recognition of the Golgi apparatus as a sensor of cellular stress and as trigger of Golgi-specific cell death pathways provide new hints to the molecular mechanisms underlying Golgi pathology.

To cover these emerging themes, this Frontiers Research Topic is organized as follows. The issue starts with a summary on Golgi functional organization in neurons (Valenzuela and Perez) and the relation of this organelle with microtubules (Sanders and Kaverina).

This is followed by pathological, genetic, and mechanistic descriptions of the major neurodegenerative diseases including Parkinson disease (Wang and Hay), Alzheimer disease by Wang and colleagues (Joshi et al.) and ALS by Atkin and colleagues (Sundaramoorthy et al.). The Research topic then focuses on Golgi fragmentation brought about by defects in vesicle biogenesis and dynamics to and through the Golgi by Lupashin and colleagues (Climer et al.) and by Schmitt-John, including those caused by defects in Golgi-derived microtubules in ALS (Haase and Rabouille) and microtubule-dependent motors in proximal SMA (Jaarsma and Hoogenraad; Wirth and Martinez-Carrera).

The third part of this issue starts by posing the hypothesis of Farhan and colleagues that cellular stress can be the cause of Golgi fragmentation, which in turn amplifies cellular stress and leads to neurodegeneration (Alvarez-Miranda et al.). This is argued by reviews on the effect of DNA damage on the Golgi by Field and colleagues (Buschman et al.) and on the role of the Golgi as a cell death trigger (Machamer).

Future studies on Golgi pathology in neurodegenerative diseases will continue to benefit not only from conceptual advances but also from new technical developements that have been gigantic since Golgi's original description of the black reaction (tissue hardening with potassium dichromate and cell staining by silver impregnation) (Mazzarello et al., 2009).

Electron microscopy has been used to unravel Golgi fragmentation (Mourelatos et al., 1996) and in particular the Golgi fragmentation into tubules and vesicles observed in degenerating motor neurons (Bellouze et al., 2014), and its resolution may be further improved in tissues prepared by high pressure freezing (Walther et al., 2013). 3D reconstructions of the Golgi and its microtubules (Marsh et al., 2001; Efimov et al., 2007) may illustrate pathological changes in their intricate connections.

Golgi fragmentation can also be monitored by live imaging (Altan-Bonnet et al., 2006), and super resolution microscopy (Betzig et al., 2006; Lippincott-Schwartz and Manley, 2009) may help refining the process. Last, system biology approches may shed light on new pathways connecting Golgi fragmentation to neurodegeneration by identifying novel gene networks (Alvarez-Miranda et al.).

However, this field faces further challenges. It will be crucial to determine whether Golgi pathology is contributory, causative or homeostatic in neurodegeneration. In particular, it is important to understand whether Golgi alterations are linked to axonal degeneration and synapse loss or dysfunction.

It will also be crucial to analyze whether Golgi pathology in each neurodegenerative disease is restricted to the neuron types that are specifically affected, i.e., motor neurons in ALS, dopaminergic neurons in PD, striatal neurons in Huntington. If so, what may be the corresponding mechanisms of vulnerability and resistance?

Furthermore, we will need to determine whether Golgi alterations in degenerating neurons impact on the function of their non-neuronal cellular neighbors, including astrocytes, microglia and Schwann cells. Can this provide a potential explanation for the non-cell autonomous disease spread observed in numerous neurodegenerative diseases?

Finally and most importantly, can our burgeoning knowledge on the molecular mechanisms of Golgi pathology in neurodegenerative diseases be translated into earlier disease diagnosis and new therapies for these severe and hitherto untreatable disorders?

## Author contributions

CR and GH prepared and wrote the manuscript.

### Conflict of interest statement

The authors declare that the research was conducted in the absence of any commercial or financial relationships that could be construed as a potential conflict of interest.
